# Transforming Growth Factor Beta (TGFβ1) and Epidermal Growth Factor (EGF) as Biomarkers of *Leishmania (V) braziliensis* Infection and Early Therapeutic Response in Cutaneous Leishmaniasis: Studies in Hamsters

**DOI:** 10.3389/fcimb.2018.00350

**Published:** 2018-10-02

**Authors:** Andrés Montoya, Lina Yepes, Alexander Bedoya, Raúl Henao, Gabriela Delgado, Iván D. Vélez, Sara M. Robledo

**Affiliations:** ^1^Programa de Estudio y Control de Enfermedades Tropicales (PECET), Facultad de Medicina, Universidad de Antioquia, Medellín, Colombia; ^2^Grupo de Investigación en Inmunotoxicología, Departamento de Farmacia, Facultad de Ciencias, Universidad Nacional de Colombia, Bogotá, Colombia

**Keywords:** growth factor, cutaneous leishmaniasis, *L. braziliensis*, EGF, TGFβI, FGF, PDGF, biomarkers

## Abstract

**Introduction:** In cutaneous leishmaniasis, the host immune response is responsible for the development of skin injuries but also for resolution of the disease especially after antileishmanial therapy. The immune factors that participate in the regulation of inflammation, remodeling of the extracellular matrix, cell proliferation and differentiation may constitute biomarkers of diseases or response to treatment. In this work, we analyzed the production of the growth factors EGF, TGFβ1, PDGF, and FGF during the infection by *Leishmania* parasites, the development of the injuries and the early response to treatment.

**Methodology:** Golden hamsters were infected with *L. (V) braziliensis*. The growth factors were detected in skin scrapings and biopsies every 2 weeks after infected and then at day 7 of treatment with different drug candidates by RT-qPCR. The parasitic load was also quantified by RT-qPCR in skin biopsies sampled at the end of the study.

**Results:** The infection by *L. (V) braziliensis* induced the expression of all the growth factors at day 15 of infection. One month after infection, EGF and TGFβ1 were expressed in all hamsters with inverse ratio. While the EGF and FGF levels decreased between day 15 and 30 of infection, the TGFβ1 increased and the PGDF levels did not change. The relative expression of EGF and TGFβ1 increased notably after treatment. However, the increase of EGF was associated with clinical cure while the increase of TGFβ1 was associated with failure to treatment. The amount of parasites in the cutaneous lesion at the end of the study decreased according to the clinical outcome, being lower in the group of cured hamsters and higher in the group of hamsters that had a failure to the treatment.

**Conclusions:** A differential profile of growth factor expression occurred during the infection and response to treatment. Higher induction of TGFβ1 was associated with active disease while the higher levels of EGF are associated with adequate response to treatment. The inversely EGF/TGFβ1 ratio may be an effective biomarker to identify establishment of *Leishmania* infection and early therapeutic response, respectively. However, further studies are needed to validate the utility of the proposed biomarkers in field conditions.

## Introduction

Leishmaniasis is a parasitic disease caused by protozoa of the genus *Leishmania* spp. which are transmitted by phlebotomies insects (Bates, 2007; Sharma and Singh, 2008). The disease is widely distributed in five continents and is endemic in low-income countries in tropical and subtropical regions (Alvar et al., 2012). The disease manifests itself in three main clinical forms known as cutaneous, mucosal, and visceral leishmaniasis. Mucosal and cutaneous leishmaniasis (ML and CL, respectively) are characterized by the presence of granulomatous lesions in the mucous and dermis, respectively (Herwaldt, 1999; Handler et al., 2015; Kevric et al., 2015), that may cause disfigurement accompanied by social stigmatization and psychological disorders, altering the economic well-being of patients (Weiss, 2008; Okwor and Uzonna, 2016). There are very few options to treat CL and ML and all have numerous disadvantages that evidence the urgent need to develop new and better therapeutic alternatives. It is known that CL is manifested as skin lesions that result from the exacerbated activation of the immune system through the recruitment of abundant monocytes, neutrophils, NK cells, and CD8+ and CD4+ T lymphocytes (Brewig et al., 2009; Hrdinka et al., 2011; Nylén and Eidsmo, 2012; Soong et al., 2012). The activation of all these cells induces a chronic inflammatory response that leads to the necrosis of the tissue and therefore to the skin damage and the appearance of ulcers (in most cases). This cutaneous lesion usually resolves after specific therapy that let the elimination of the antigenic stimulus and therefore, the resolution of the inflammatory response and repair of the damaged tissue (McGwire and Satoskar, 2014; Copeland and Aronson, 2015; de Menezes et al., 2015; Aronson et al., 2016).

Although the immune response to *Leishmania* has been described during infection and disease, this immune response during and after treatment has been poorly described. To date, no information is available about the production of dermal and epidermal growth factors during *Leishmania* infection or concerning the healing of lesions caused by *Leishmania* spp. Studies carried out with other types of ulcers reported that during healing, the epidermal tissue is formed after transformation of the dermis into fibrous tissue (scarring) accompanied by an increase in NK cells, a decrease in circulating T cells with depletion of CD8+ T cells, and a decrease of CD4+ T cells with a decrease in the production of IFNγ (Lakhal-Naouar et al., 2015) and expression of dermal and epidermal growth factors (Kiwanuka et al., 2012).

Growth factors are polypeptides that stimulate cell proliferation, migration, and differentiation as well as survival and apoptosis (Bennett and Schultz, 1993). The most important growth factors in this process of tissue repair are fibroblast growth factor (FGF), platelet derived growth factor (PDGF), transforming growth factor beta 1 (TGFβ1) and alpha (TGFα), epidermal growth factor (EGF), connective tissue growth factor (CTGF), and vascular endothelial growth factor (VEGF; (Robson, 1991; Steed, 1997)). The source and function of these growth factors are summarized in Table 1. While some observations suggest an important role for TGFβ during parasite establishment in the early stages of human CL (Barral et al., 1995) and treatment failure in patients with visceral leishmaniasis (Elmekki et al., 2016) there seem to be no reports of studies for the other growth factors in the context of leishmaniasis.

**Table 1:** Source and function of growth factor involved in repair of the skin.

**Growth factor^a^**	**Source**	**Function**	**References**
FGF	Endothelial cells, fibroblast, T lymphocytes	Angiogenesis, prolipheration, and migration of keratinocytes	Andres et al., 2013
PDGF	Macrophages, endothelial cells, smooth muscle, and platelets	Fibroblast and macrophages activation for the subsequent production of prostaglandins, thromboxanes and leukotrienes that in turn, increase chemotaxis and vasodilatation to promote angiogenesis, extracellular matrix deposition, and remodeling of the damaged tissue	Judith et al., 2010
TGFβ1	Macrophages, dendritic cells, fibrocytes, chondrocytes, and lymphocytes	Stimulation of extracellular matrix synthesis	Douglas, 2010; Ramirez et al., 2013
EGF	Macrophages and keratinocytes	Stimulation of mitosis and migration of keratinocytes and fibroblasts, formation of granulation tissue, contraction of wounds, and extracellular matrix deposition	Singla et al., 2012; Shen et al., 2017
CTGF	Fibroblast	Stimulation of prolipheration and chemiotaxis of GF, formation of granulation tissue, re-epithelialization, extracellular matrix remodelation, and connective tissue formation	Barrientos et al., 2008; Ponticos, 2013
TGFα	Keratinocytes, macrophages, fibroblast, and lymphocytes	Keratinocyte migration and re-epithelialization	Singh and Coffey, 2014
VEGF	Neutrophils, fibroblast, macrophages, endothelial cells, muscle cells	Blood vessel repair and granulation tissue formation	Johnson and Wilgus, 2014
KGF	Keratinocytes	Proliferation, migration, and morphogenesis of epithelial cells	Dörr et al., 2002

a *FGF, fibroblast growth factor; PDGF, platelet derived growth factor; TGFβ1, transforming growth factor beta 1; EGF, epidermal growth factor; CTGF, connective tissue growth factor; TGFα, transforming growth factor alpha; VEGF, vascular endothelial growth factor*.

Based on the important role of growth factors during healing and tissue repair, this study aimed to identify the expression levels of EGF, FGF, PDGF, and TGFβ1 during: (i) infection by *Leishmania (V) braziliensis*, (ii) development of CL, and (iii) early response to treatment using the experimental model for CL in the golden hamster (*Mesocricetus auratus*). An useful RT-qPCR method to quantify EGF, FGF, PDGF, and TGFβ1 expression was also standardized.

## Materials and methods

### Compounds

Propantheline bromide (CAS #50-34-0), limonin (CAS #1180-71-8), nomilin (CAS #1063-77-0), azadirachtin (CAS #11141-17-6), cryptolepine hydrate (CAS #480-26-2), glycyrrhizin (CAS #1405-86-3), and oleanolic acid (CAS #508-02-1) were purchased at Sigma Aldrich (St Louis, MO, USA) and were used as active pharmaceutical ingredients (API) for new formulations. Meglumine antimoniate (MA) (Sanofi-Aventis Bogotá, Colombia) was used as a reference antileishmanial drug.

### Parasites

Green fluorescent protein (GFP)-transfected *L*. *(V) braziliensis promastigotes* (MHOM/CO/88/UA301-EGFP) were maintained in biphasic Novy-MacNeal-Nicholle (NNN) medium phosphate buffer saline (PBS) enriched with glucose at pH 6.9.

### Formulations

An oil-in-water (O/W) semisolid emulsion was prepared which served as the matrix in which the API were embedded. The designed formulations were as follows: 0.5% propantheline bromide, 2.4% limonin, 2.2% nomilin, 1.1% azadirachtin, 1.4% cryptolepine hydrate, 5% glycyrrhizin, and 2.5% oleanolic acid. The concentration of the API was selected at convenience, according to the availability of the compound and previous results of the antileishmanial activity obtained *in vitro*.

### Animals

Eighty-six week-old hamsters, male and female, with an average weight of 180 g (160–200 g) were used. Hamsters were maintained in specific pathogenic-free conditions in an animal facility at Universidad de Antioquia (Medellín, Colombia), housed in transparent cages in groups of three or four animals per cage, during the study.

### Experimental infection

Previous anesthesia with a mixture of ketamine (40 mg/kg) and xylazine (5 mg/kg), hamsters were injected in the dorsum with 5 × 10^8^ promastigotes of *L. braziliensis* in the stationary phase of growth (day 6 in culture) in 100 μl PBS. After 15 and 30 days post-infection (ID15 or ID30, respectively), one group of eight hamsters each were humanely sacrificed. Skin biopsies from the inoculation site were obtained and stored in RNA later for the RNA extraction. The remaining hamsters were kept under observation until the development of ulcers. Skin biopsies of four uninfected hamsters were obtained and they corresponded to biopsies obtained before infection day (ID0).

### Therapeutic response

Four weeks after the infection, ulcers were formed, and treatment with each of the formulations had started. For this, the hamsters were randomly distributed in eight groups with eight hamsters each. Each group was treated topically during 20 days with 40 mg/day of one of the following formulations: 0.5% propantheline bromide, 2.4% limonin; 2.2% nomilin, 1.1% azadirachtin, 1.4% cryptolepine hydrate, 5% glycyrrhizin, and 2.5% oleanolic acid; or intramuscular MA (120 mg/kg/10 days). The hamsters were monitored daily during the study recording any change in behavior or death. The area of the injuries was measured before treatment (TD0) and 90 days after completion of the treatment (PTD90), which corresponded to the end of the study. The effectiveness of each treatment was determined according to the area of the lesion at PTD90 with respect to the size of the lesion before treatment. The response to the treatment was classified as *cure* (if healing of 100% of the area of the lesion did occur) or *improvement* (if the area of injury decreased more than 20%). On the contrary, the outcome was classified as *failure* (if the area of injury increased) or as *relapse* (if reactivation of the injury appeared after an initial cure). At the end of the study, hamsters were sacrificed humanely in a CO_2_ chamber and necropsied. Skin samples for parasite load analysis were obtained. Prior to treatment (TD0) and on day 7 of treatment (TD7), scraping samples were taken from the lesions and deposited in RNA later (for RNA extraction).

### RNA extraction

The RNA from the skin samples was extracted using Trizol^®;^ (Invitrogen) following the manufacturer's instructions and then quantified in a Nanodrop 1000 (Thermo scientific).

### Retrotranscription

One hundred grams of RNA were treated with 1 μl *DNase* I (Fermentas), 1 μl of buffer, and 8 μl of nuclease free water; the mixture was incubated in a thermocycler PTC 100^TM^ (MJ research) for three cycles: 30 min at 37°C, 5 min at 4°C, and 10 min at 65°C. Using the maximum first strand cDNA synthesis kit, the RNA was transcribed into cDNA, following the manufacturer's instructions: 4 μl master mix reaction, 2 μl RT enzyme mix, 2 μl RNA *DNase* I treated, and 12 μl water were mixed and incubated in the PTC 100^TM^ (MJ research) thermocycler for three cycles: 10 min at 25°C, 15 min at 50°C, and 5 min at 85°C.

### Growth factor expression

Specific primers for EGF, PDGF, FGF, and TGFβ1, as well as fluorescent FAM-labeled hydrolysis probes, were designed. The sequences for each forward (Fw) and reverse (Rv) primer and probe used were as follows:

*FGF*,Fw,5′-GTGTCAAGGCTGCTAGGTTT-3′, Rv,5′-ACACATTGTATCCATCCTCAA-3′ and probe 5′-TCGCCTCACTTCGATCCCG-3′; *EGF*,Fw,5′-CAGAACAAAGCCAGAAAATC-3′, Rv,5′-CTGCAAGTACGTTCGTTTAACT-3′ and probe 5′-AGACTCGCGTTGCAAGGCG-3′; *PDGF*,Fw,5′-GGCTCGAAGTCAGATCCATA-3′,Rv,5′-CTTCTCCGGCACATGCTTAA-3′and probe 5′-TGGAGACAAGCCTGAGAGCC-3′;TGFβ1,Fw,5′-AGCCTGGACACACAGTACAGTA-3′,Rv,5′-CTTGCGACCCACGTAGTAC-3′and probe 5′-AACACAACCCGGGTGCTTC-3′;γActin,Fw,5′-ACAGAGAGAAGATGACGCA-3′,Rv,5′-GCCTGAATGGCCACGTAC-3′and probe 5′-TTGAAACCTTCAAATGACGCA-3′.

The reaction of amplification was carried out with 1 μl of the cDNA and using the following protocol: 600 s at 95°C, 40 cycles of 15 s at 95°C, and 60 s at 60°C in a Smart Cycler II (Cepheid, Sunnyvale, CA, USA). The efficiency of the amplification reaction was determined using the LinReg program and the expression levels were calculated using the ΔΔCT method, comparing the levels of expression for PI15, PI30 with respect to healthy skin before infection and TD7 with respect to TD0. Additionally, the levels of induction were compared in terms of clinical outcome, in terms of cure, improvement, relapse, or failure.

### Parasite load

The parasitic load on the skin biopsy samples taken at the end of the study (PRD90) was determined by RT-qPCR using the Vero 1-step RT-qPCR SYBR Green kit, a 123 bp fragment of the DNA polymerase I gene from Leishmania was amplified. To do this, 20 ng of RNA, 12.5 μl the mix, and 100 ng of the each primer Fw 5′-TGAGCGCATCGAGTACCT-3′ and Rv 5′-TCCCGCTTGCCATCCTC-3′, with a volume adjusted to 25 μl using nuclease-free water, were used to carry out the reaction with the Smart Cycler II (Cepheid, Sunnyvale, CA, USA): 50°C 15 min, 95°C 15 min and 40 cycles 95°C 15 s, 60°C 20 s and 72°C 20 s, a final cycle 72°C 300 s and a melting curve between 60 and 95°C. Absolute quantification was performed using a standard logarithmic scale from 1 to 1 million parasites.

### Ethical aspects

All the procedures were approved by the Ethics Committee for Animal Experimentation of the Universidad de Antioquia (Act No. 91 of 2014).

### Statistical analysis

The differences between the parasitic loads according to the clinical results were determined by a one-way ANOVA and a Tukey's test for multiple comparisons. The differences in the expression of growth factors between ID15 and ID30 vs. ID0 and between TD0 and TD7 were determined by two-way ANOVA and Bonferroni's test for multiple comparisons. In addition, differences in expression levels according to the clinical results were also obtained by two-way ANOVA and Bonferroni's test.

## Results

### Lesion progression in hamsters

**Figure 1:**
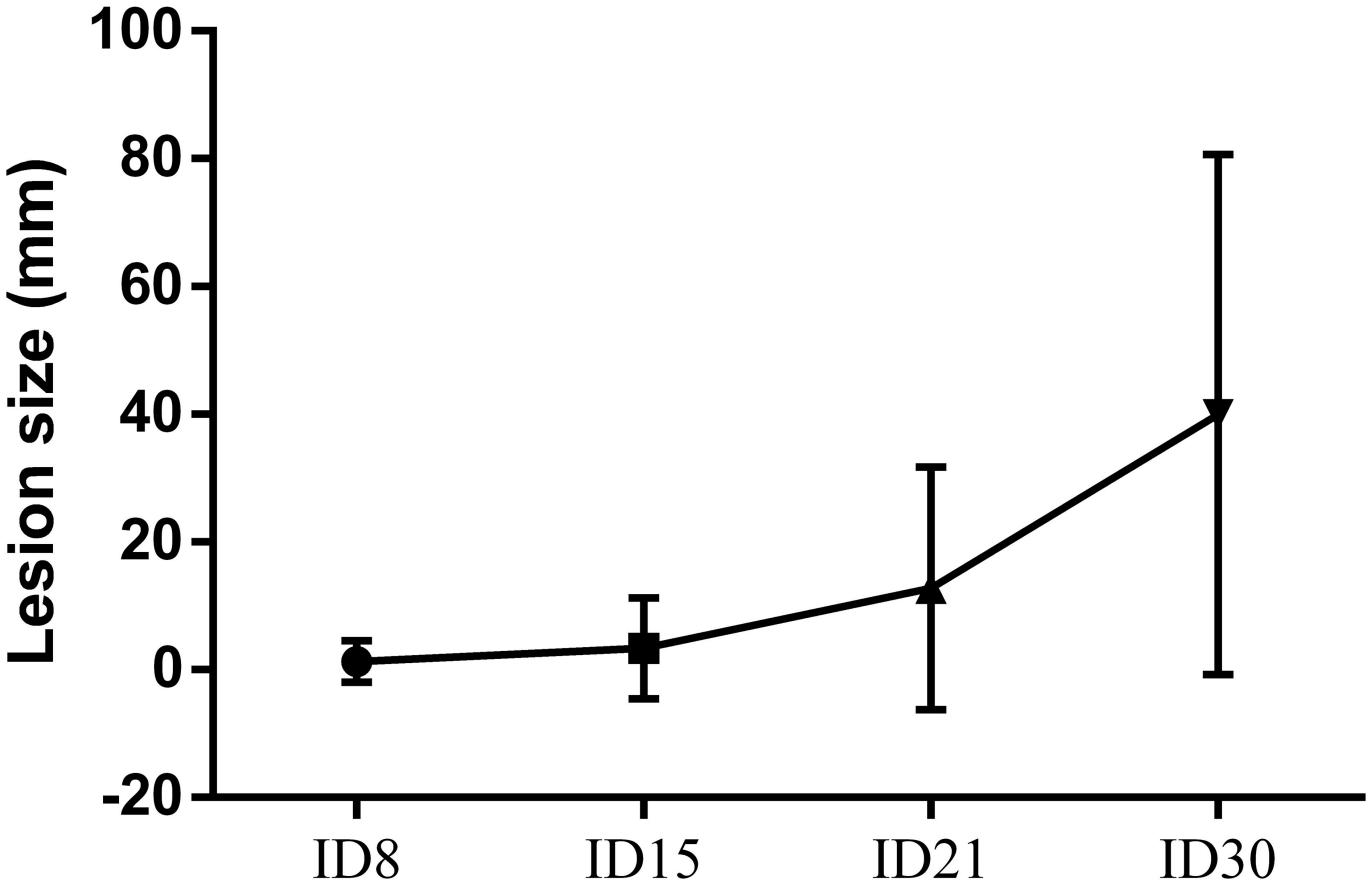
Cutaneous leishmaniasis progression. Quantification of lesion area in in golden hamsters experimentally infected with 5 × 10^8^ promastigotes of *L. (V) braziliensis*. Data are presented as the mean ± SD of 24 hamsters at days 8, 15, 21, and 30 after infection.

After intradermal injection of promastigotes of *L. (V) braziliensis* promastigotes in dorsum, hamsters were monitored weekly for any sign of induration or skin damage at the site of inoculation. The diameter of the injury increased in size every week. In this way, the average size of the injuries was 1,29 ± 3.29 mm^2^ at ID8, 3.35 ± 7.92 mm^2^ at ID15, 12.75 ± 19 mm^2^ at ID21, and 39.93 ± 40.73 mm^2^ at ID30. The lesion on the skin was evidenced first as a nodule and then as an ulcer. The induration varied from mild in the second week post-infection to exacerbated in the 4 week after inoculation (Figure 1).

### Expression of PDGF, EGF, FGF, and TGFβ1 associated with the process of infection by *L. (V) braziliensis* in the hamster model for cutaneous leishmaniasis

**Figure 2:**
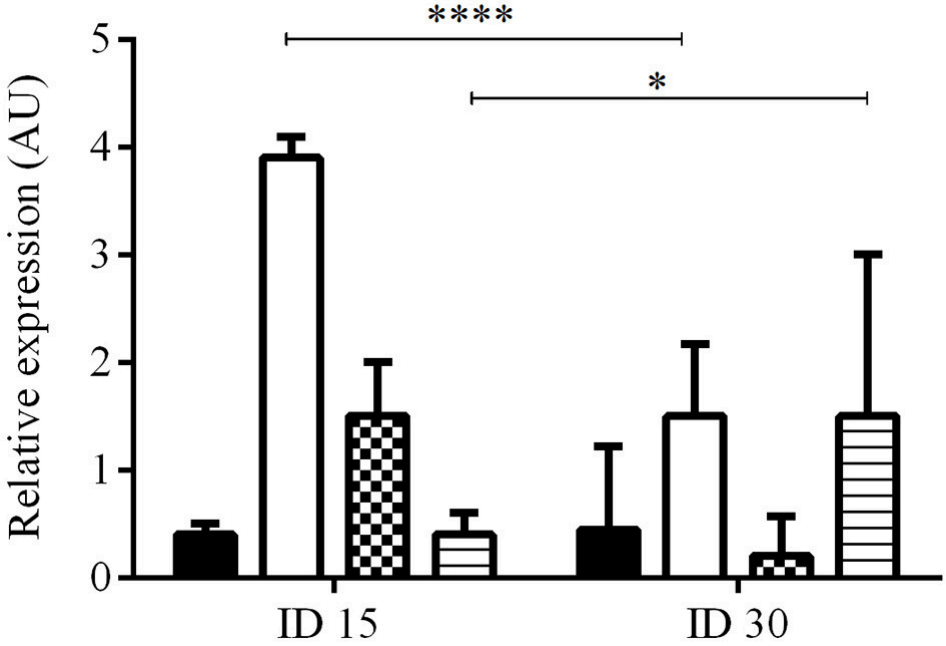
Relative expression of growth factors during infection and development of injury. The figure shows the relative expression at ID1 (*n* = 8) and ID30 (*n* = 8) vs. before infection (ID0) calculated by the ΔΔCT method. PDGF (black), EGF (white), FGF (pattern), and TGFβ1 (lines). ****p < 0.0001; *p < 0.0442.

The expression levels of PDGF, EGF, FGF, and TGFβ1 were calculated by the ΔΔCT method and expressed as the mean ± SD of the number of times that each factor was induced with respect to healthy skin before infection (ID0), during the development of the lesion (ID15 and ID30). Expression levels lower than the constitutive gene were found for the PDGF, EGF, TGFβ1, and FGF before infection, and these basal levels were used for the calculation of ΔΔCT. Infection by *L. (V) braziliensis* induced the expression of all the growth factors in ID15, with levels of induction with respect to healthy hamsters of: PDGF 0.4 ± 0.1, EGF 3.9 ± 0.2, FGF 1.1 ± 0.5, and TGFβ1 0.4 ± 0.2. In ID30, PDGF was expressed in 3/8 hamsters with an expression value of 0.6 ± 0.1; EGF and TGFβ1 were expressed in all hamsters with an expression value of 1.5 ± 0.7 and 1.6 ± 0.5, respectively. Lastly, FGF was expressed in 2/8 hamsters and its expression value was 0.2 ± 0.0. The differences in EGF and TGFβ1 expression levels between ID15 and ID30 were statistically significant with *p* < 0.0001 and < 0.0442, respectively. On the contrary, the differences between PDGF and FGF were not significant (Figure 2).

### Expression of PDGF, EGF, FGF, and TGFβ1 in response to treatment

**Figure 3:**
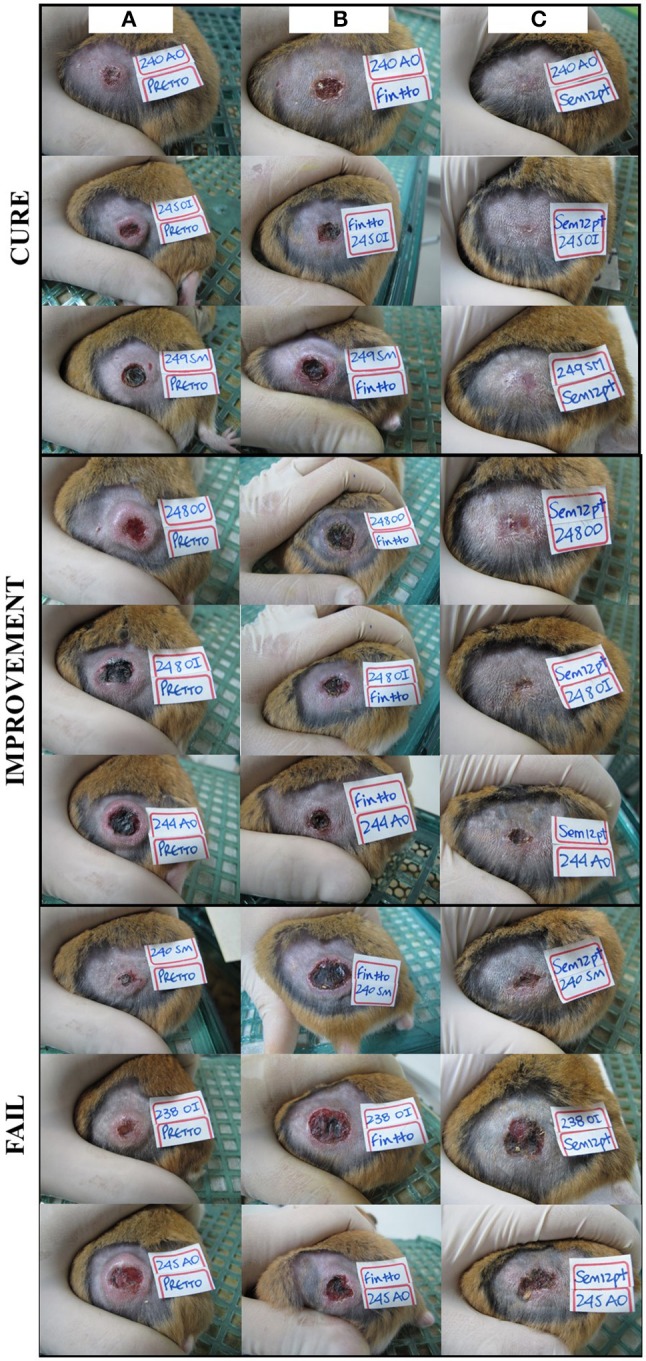
Treatment progress of cutaneous leishmaniasis in hamsters experimentally infected with *L. (V) braziliensis***. **A representative photograph of lesion, **(A)** before treatment, **(B)** end of treatment, and **(C)** 90 days post-treatment. Note the complete re-epithelialization of the skin in cured hamsters, the decrease in size of the injury during improvement or the increases in the size of the injury during fail to treatment.

**Figure 4:**
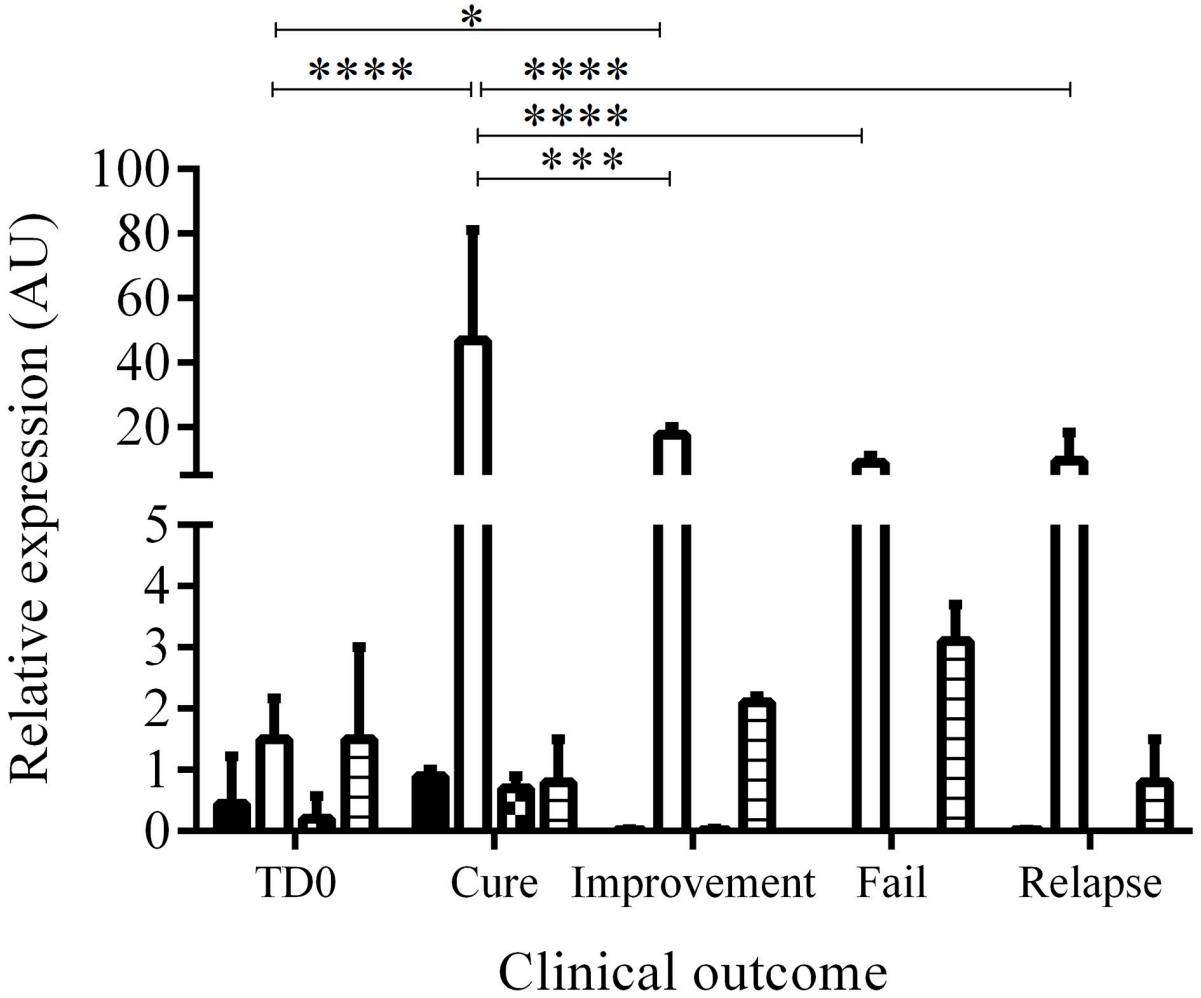
Relative expression of growth factors before and during treatment. The figure shows the induction of growth factors calculated by the ΔΔCT between TD0 and TD7 method according to the clinical outcome. Bars represent the mean value ± SD. PDGF (black), EGF (white), FGF (square), and TGFβ1 (lines). *p < 0.0413; ***p < 0,0003; ****p < 0.0001, cured (*n* = 10), improvement (*n* = 24), fail (*n* = 27), relapse (*n* = 3).

Of 64 hamsters treated with the different compounds and followed up for 3 months, 10 hamsters (15.6%) cured, 24 hamsters (37.5%) showed improvement of their ulcers, 27 hamsters (42.2%) failed, and 3 hamsters (4.6%) relapsed. The appearance of the lesions according to the clinical outcomes after treatment are shown in Figure 3. The levels of expression of PDGF, EGF, FGF, and TGFβ1 at TD7 were compared with respect to the levels before treatment (TD0) using the ΔΔCT method. In the cured hamsters, the expression levels increased 0.9 ± 0.1 times for PDGF, 46.9 ± 34.2 for EGF, 0.7 ± 0.2 for FGF, and 0.8 ± 0.7 for TGFβ1 (Figure 4). In the hamsters with improvement of their ulcers, the expression levels increased 0.011 ± 0.017 times for PDGF, 17.5 ± 2.4, for EGF, 0.01 ± 0.03 for FGF, and 2.1 ± 0.1 for TGFβ1. In turn, in the hamster that relapsed, the levels for PDGF were 0.012 ± 0.01, 9.5 ± 8.8 for EGF, 0.8 ± 0.7 for TGFβ1, and FGF was not detected. Finally, in the hamsters that failed to the treatment, the expression levels for EGF and TGFβ1 increased 8.9 ± 2.1 and 3.1 ± 0.6 times, respectively, while PDGF and FGF were not detected. Significant differences in expression were only observed in EGF expression between cured hamsters vs. TD0 (*p* < 0.0001), improvement vs. TD0 (*p* < 0.0413), cured vs. improvement (*p* < 0.0003), cured vs. failure (*p* < 0.0001), and cured vs. relapsed (*p* < 0.0001) (Figure 4).

Only EGF and TGFβ1 showed expression levels >1. When compared the proportion of EGF vs. TGFβ1 we found that the EGF was expressed 58.6 times more than the TGFβ1 in cure and 8.3 in improvement while in relapses and fails the proportion was 11.8 and 2.9, respectively. Although the EGF increases during treatment, the increase is lower in relation to a poor therapeutic response. Thus, for example, the EGF was expressed 2.68 more times in cure vs. improvement, 4.93 times more in cure vs. relapse and 5.26 times more in cure vs. failure. In a similar way, the TGFβ1 was expressed 3.9 times more in failure vs. cure, 2.6 times more in improvement vs. cure.

The effect of each treatment in the expression of each growth factor in terms of increasing, reducing, or no changes with respect to the clinical outcome is shown in Table 2. The treatment with meglumine antimoniate and propantheline bromide, both drugs associated with cure of CL in hamster, showed the major increases in the expression of EGF.

**Table 2:** Induction of growth factor expression by treatment.

**Treatment**	**PDGF**				**EGF**				**FGF**				**TGF**β**1**			
	**C**	**I**	**F**	**R**	**C**	**I**	**F**	**R**	**C**	**I**	**F**	**R**	**C**	**I**	**F**	**R**
Propanteline bromide	++	=	=	**–**	++++	++	++	**–**	++	**–**	+	**–**	+	++	++	**–**
Limonin	–	+	+	–	–	++	++	–	–	+	–	–	–	++	++	–
Nomilin	–	–	–	–	–	++	+	–	–	–	–	–	–	++	++	–
Azadirachtin	–	–	–	–	–	++	-	–	–	–	–	–	–	+	++	–
Cryptolepine hydrate	–	–	+	–	–	++	-	–	–	–	+	–	–	++	-	–
Glycyrrhizin	–	+	–	–	–	++	+	–	–	–	–	–	–	+	++	–
Oleanolic acid	–	–	+	–	–	++	++	–	–	–	–	–	–	+	++	–
Meglumine antimoniate	++	=	=	=	++++	++	++	=	++	**–**	=	=	+	++	++	++

*The effect of each treatment on the expression of each factor was determined according to the changes in the expression level as increase (+), no change (= ), or not effect (–). C, cure; I, improvement; F, failure; R, Relapse. +, < 1 increase fold; ++, >1 y < 5 increase fold; ++++, >40 increase fold, n = 8, each group*.

### Parasite load during infection by *L. (V) braziliensis* and treatment in the golden hamster (*mesocricetus auratus*)

**Figure 5:**
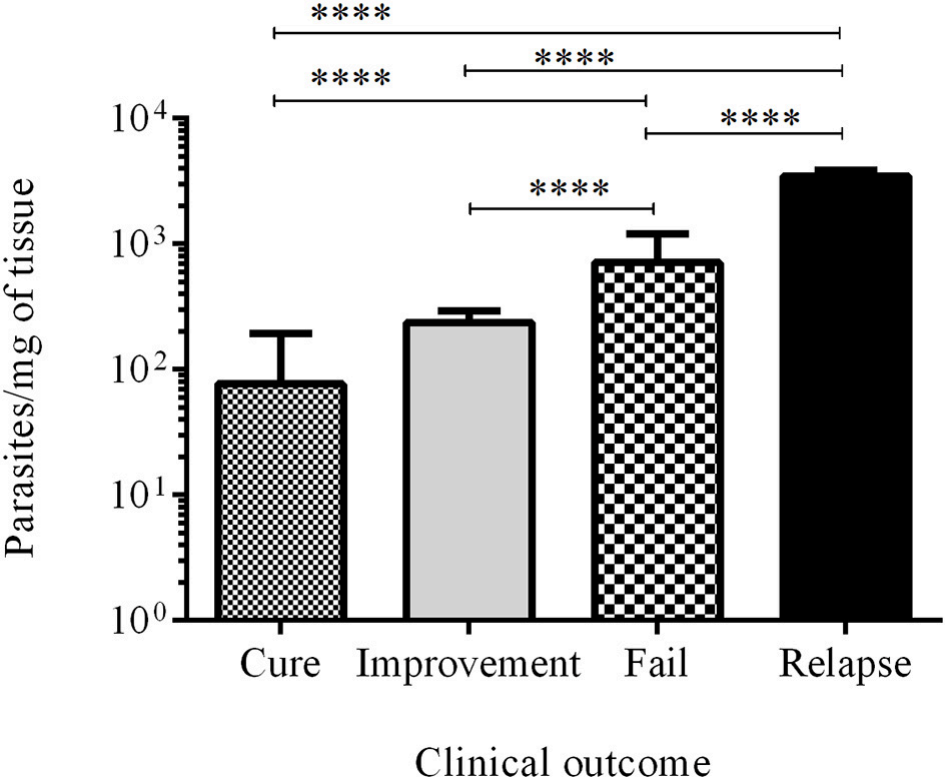
Parasite load. Bars represent the mean value ± SD of the number of parasites per mg of tissue in cured hamsters (white bar), improvement (gray bar), failure (square bar), and relapse (black bar). ****p < 0.0001.

The number of parasites in the injury during infection (ID15 and ID30) and at the end of the study (PTD90) was measured by RT-qPCR and was expressed as parasites per mg of tissue. The parasite load in the 10 cured hamsters was 76.8 ± 115.8, while in the 24 animals with clinical improvement the parasite load was 238.0 ± 57.0. In turn, in the 27 animals classed as failures, the parasite load was 710.4 ± 484.5, while in the 3 hamsters that relapsed the parasitic load was 3478.0 ± 388.6. Differences were statistically significant between cured vs. fail, cured vs. relapsed, improvement vs. failure, improvement vs. relapse, and failure vs. relapsed. In all cases, the p value was < 0.0001 (Figure 5).

## Discussion

For several decades, there have only been five drugs used for the treatment of leishmaniasis. Although these are still effective, they are far from ideal due to factors such as costs, duration of treatment, toxicity, and the appearance of resistant strains (Oliveira et al., 2011; Monge-Maillo and López-Vélez, 2013; Wolf Nassif et al., 2017). The intensive work done in the last 15 years in the search for alternative treatments of leishmaniasis has allowed for the identification of numerous candidate molecules or compounds. Some of these have advanced in their development until the stage of clinical evaluation. However, only a few are considered as real candidates for the development of medicines to date. Many candidates fail because of the poor effectiveness demonstrated in phase II clinical trials (Singh et al., 2012). The extremely high cost of clinical trials is a bottleneck in the development of new drugs; therefore, it is necessary to identify markers that allow for early identification of the response to the treatment that is being evaluated (Frank and Hargreaves, 2003; Zwierzina, 2008).

Although multiple studies have analyzed susceptibility and resistance genes associated to the development of the lesions (Castellucci et al., 2012; Sohrabi et al., 2013; Abdoli et al., 2017), the role of EGF, FGF, and PDGF in the establishment of the infection, development of the ulcer, and in early response to treatment has not been established. In contrast, although the TGFβ1 has been related to susceptibility and chronicity of the infection (Balak et al., 1992; Barral-Netto and Barral, 1994; Barral et al., 1995; Bogdan and Röllinghoff, 1998; Nieto et al., 2011; Hejazi et al., 2012; Rodrigues et al., 2014; Bhattacharya et al., 2016), its role has not been described in the context of early therapeutic response. In this work, we described the dynamics of the production of EGF, FGF, PDGF, and TGFβ from initial infection and manifestation of skin lesions to the early stages of response to different treatment compounds. We demonstrated that the expression of PDGF, EGF, FGF, and TGFβ1 was induced at ID15 during the establishment of the experimental infection by *L. (V) braziliensis*. This result suggests that, during infection, the cells are activated to control the tissue damage as a result of inflammation (Mast and Schultz, 1996). At ID30 when the infection is already established and the ulcer is manifested, the expression of growth factors changes drastically, not only in the profile of factors, but also in the level of induction (Mast and Schultz, 1996). FGF and PDGF were no longer expressed in more than half of the animals, but EGF and TGFβ1 showed an interesting change reversing their induction levels by increasing TGFβ1 and decreasing EGF.

The decreased expression of PDGF and FGF correlates with the appearance of lesions by ID30. It has been confirmed in the hamster model for CL that infection by *Leishmania* generates the activation of the Nlrp3 inflammasome with the subsequent production of IL1β (Lima-Junior et al., 2013), and this cytokine may negatively regulate the expression of PDGF (Barrientos et al., 2008; Mundy, 2009). In the case of FGF and other growth factors, such as TGFα, KGF, and VEGF, it has been shown in the experimental CL hamster model that, in some types of ulcers induced by drugs or anti-tumor therapies, the decrease in proinflammatory cytokines such as IL1β and TNFα positively regulates the expression of these factors (Araújo et al., 2015). When the inflammatory process perpetuates, it has a negative regulatory effect on these two growth factors, PDGF and FGF, in some cases, halting expression and in other cases, reducing it. Another explanation for the reduction in the expression of these growth factors is that the macrophages, keratinocytes, endothelial cells, and fibroblasts involved in the production of growth factors in the infected and inflamed tissue are in a context where their functions of survival, proliferation, cell differentiation, and angiogenesis have been regulated by the need to eliminate the parasite.

Interestingly, TGFβ1 tripled its expression in 8/8 hamsters during infection. In addition, other studies have associated the early expression of this factor and its increased expression levels with the regulation of the immune response, allowing for the establishment of infection and development of ulcers (Balak et al., 1992; Barral-Netto and Barral, 1994; Barral et al., 1995; Mougneau et al., 2011; Hejazi et al., 2012; Nylén and Eidsmo, 2012; Rodrigues et al., 2014; Bhattacharya et al., 2016), since TGFβ1 has the capacity to inhibit the Th1 response affecting the production of IFNγ, IL12, and key cytokines in the control and elimination of the parasite (Reed, 1999). On the other hand, the expression of EGF decreased with respect to ID15, reducing by half in 8/8 animals. This growth factor is very important for the proliferation of keratinocytes, which are essential for the establishment of a Th1 immune response that favors the control of the infection (Steed, 1997; Eming et al., 2007; Kiwanuka et al., 2012; Pikuła et al., 2015). Its increase at ID15 is likely to be an attempt to favor the Th1 response. However, when the immune response is regulated by TGFβ1 and the infection is effectively established at ID30, its expression is negatively regulated, due to the fact that the macrophages and fibroblasts, as its main sources, are in a context in which the production of EGF is diminished.

When analyzing the early expression of growth factors as biomarkers for the clinical outcome of our experimental model, we initially found differences in the expression profile of these factors. Thus, in the 3 animals that relapsed after treatment, the EGF was expressed at higher levels that TGFβ1; nevertheless, since it is only a group of three hamsters, it limits the comparisons with the other groups. In turn, in hamsters in which the treatment failed, the expressed factor profile involved EGF, and TGFβ1. In turn, in the cured hamsters and those that showed improvement of their injuries, all growth factors, even FGF, were expressed.

When comparing the production profile of growth factors in fetal and adult fibroblasts, differences have been correlated with the healing and the re-epithelialization of injuries and a better appearance of the scars, which explains why fetal scarring leaves scarcely imperceptible scars (Broker et al., 1999). However, the presence of several factors is necessary in order to repair damaged tissue efficiently (Papanas and Maltezos, 2007; Buchberger et al., 2010). Some clinical trials have evaluated the use of one or several growth factors such as PDGF, EGF, and FGF for the treatment of chronic ulcers, showing good results when used individually but with better results when used in combination (Robson, 1991; Mast and Schultz, 1996; Singla et al., 2012).

On the other hand, the profile of the growth factors in our model allowed us to predict a positive clinical result of the treatment at an early stage, as well as the levels of induction of the different growth factors. In the case of FGF and PDGF, since their expression is not constant in all hamsters, it is difficult to conclude that the levels of induction of these factors are a good biomarker for early therapeutic response. However, the benefit of knowing the behavior of the expression of these growth factors and that their early induction depends on the treatment will allow for future evaluations in our animal model to identify candidates for medications that possess not only antileishmanial potential but also scarring potential.

In the case of TGFβ1, its role in pathogenesis is well described. There is an increase in the levels of TGFβ1 in peritoneal macrophages infected with the parasites (Balak et al., 1992). In human biopsies from active lesions in both the cutaneous form and the mucosal form of the disease, the presence of this factor is also detected (Barral et al., 1995), which is related to one of the functions of TGFβ1 in the regulation of the immune response allowing for the parasite to replicate (Abdoli et al., 2017). TGFβ1 appears to be a candidate biomarker for pathogenesis and for a negative therapeutic response to treatment (failure and relapse). Although the differences in the levels of expression were not statistically significant, the tendency is to increase in production when there is failure or relapse, and decrease in production when curing or improvement occur.

EGF showed significant differences in the levels of induction on TD7 with respect to TD0 and difference in the expression with respect to the clinical result among the cured animals and all the other clinical outcomes. EGF plays a very important role in the process of cell proliferation, activation, and re-epithelialization (Barrientos et al., 2008). It increases in acute wounds (Shen et al., 2017) and is found to decease in chronic wounds (Mast and Schultz, 1996). In the case of CL, we confirm low levels of EGF before treatment, because it is a chronic injury. However, by implementing a treatment that induces healing, we find that the level of EGF expression increases, in comparison to the treatments that produce improvement, failure, or relapse. Notoriously, propantheline bromide and MA, which were medications associated with healing, showed a similar profile. These results suggest that this profile can help predict a curative response during treatment follow-up.

This study focused on the search for biomarkers in leishmaniasis and allowed us to establish the role of multiple cytokines in the process of infection and in pathogenesis as well as in the candidates for biomarkers related to the immune response profile in visceral leishmaniasis (Portela et al., 2018). In the case of canine visceral leishmaniasis, a recent study identified some proteins as biomarkers of the therapeutic response in dogs. Nevertheless, none of these proteins were growth factors or were related to the activation of growth factors (Martinez-Subiela et al., 2017). This is most likely because the samples used for monitoring the therapeutic response biomarkers were taken 1 month after the end of the treatment, when the parasite is likely to have been eliminated. In this case, the samples would not indicate the effectiveness of the treatment early but would confirm whether the treatment was effective. Our results allow us to implement in our experimental model a rapid measurement of the therapeutic response to treatment and define the optimal dose or frequency, thus obtaining better therapeutic success with the new candidates that are being tested *in vivo* for the treatment of CL.

## Conclusion

A differential profile of EGF, TGFβ1, PDGF, and FGF expression was observed during the establishment of *L. (V) braziliensis* infection and response to treatment in our experimental CL model. Higher induction of TGFβ1 are more associated with active disease (infection and failure or relapses after treatment) while the higher levels of EGF are associated with adequate response to treatment. Thus, the inversely EGF/TGFβ1 ratio may be an effective biomarker to identify establishment of *Leishmania* infection and early therapeutic response, respectively. However, further studies are needed to validate the utility of these growth factors as biomarkers in the pathogenesis of human CL and response to treatment under field conditions.

## Author contributions

AM participated in the design of the project, carried out the trials, analyzed the results, and participated in writing of the manuscript for publication. SR was in charge of the project and the analysis of results, and participated in writing of the manuscript. LY, AB, and RH helped in performing the experiments. GD and IV participated in the analysis of results and in the writing of the manuscript.

### Conflict of interest statement

The authors declare that the research was conducted in the absence of any commercial or financial relationships that could be construed as a potential conflict of interest. The reviewer AIM and handling editor declared their shared affiliation at time of review.
